# A risk calculator to inform the need for a prostate biopsy: a rapid access clinic cohort

**DOI:** 10.1186/s12911-020-01174-2

**Published:** 2020-07-03

**Authors:** Amirhossein Jalali, Robert W. Foley, Robert M. Maweni, Keefe Murphy, Dara J. Lundon, Thomas Lynch, Richard Power, Frank O’Brien, Kieran J. O’Malley, David J. Galvin, Garrett C. Durkan, T. Brendan Murphy, R. William Watson

**Affiliations:** 1 Conway Institute of Biomolecular and Biomedical Research, University College Dublin, Dublin, Ireland; 2 UCD School of Medicine, University College Dublin, Dublin, Ireland; 3 UCD School of Mathematics and Statistics, University College Dublin, Dublin, Ireland; 4 Insight Centre for Data Analytics, University College Dublin, Dublin, Ireland; 5 Department of Urology, St. James University Hospital, Dublin, Ireland; 6 Department of Urology, Beaumont Hospital, Dublin, Ireland; 7 Department of Urology, University Hospital Waterford, Waterford, Ireland; 8 Department of Urology, Cork University Hospital, Cork, Ireland; 9 Department of Urology, Mater Misericordiae University Hospital, Dublin, Ireland; 10 Department of Urology, St Vincent’s University Hospital, Dublin, Ireland; 11 Department of Urology, University Hospital Galway, Galway, Ireland; 12 Department of Urology, University Hospital Limerick, Limerick, Ireland

**Keywords:** Biopsy, Prostate Cancer, Decision-making, Risk calculator, Binary logistic regression, cross-validation, Rshiny

## Abstract

**Background:**

Prostate cancer (PCa) represents a significant healthcare problem. The critical clinical question is the need for a biopsy. Accurate risk stratification of patients before a biopsy can allow for individualised risk stratification thus improving clinical decision making. This study aims to build a risk calculator to inform the need for a prostate biopsy.

**Methods:**

Using the clinical information of 4801 patients an Irish Prostate Cancer Risk Calculator (IPRC) for diagnosis of PCa and high grade (Gleason ≥7) was created using a binary regression model including age, digital rectal examination, family history of PCa, negative prior biopsy and Prostate-specific antigen (PSA) level as risk factors. The discrimination ability of the risk calculator is internally validated using cross validation to reduce overfitting, and its performance compared with PSA and the American risk calculator (PCPT), Prostate Biopsy Collaborative Group (PBCG) and European risk calculator (ERSPC) using various performance outcome summaries. In a subgroup of 2970 patients, prostate volume was included. Separate risk calculators including the prostate volume (IPRCv) for the diagnosis of PCa (and high-grade PCa) was created.

**Results:**

IPRC area under the curve (AUC) for the prediction of PCa and high-grade PCa was 0.6741 (95% CI, 0.6591 to 0.6890) and 0.7214 (95% CI, 0.7018 to 0.7409) respectively. This significantly outperforms the predictive ability of cancer detection for PSA (0.5948), PCPT (0.6304), PBCG (0.6528) and ERSPC (0.6502) risk calculators; and also, for detecting high-grade cancer for PSA (0.6623) and PCPT (0.6804) but there was no significant improvement for PBCG (0.7185) and ERSPC (0.7140). The inclusion of prostate volume into the risk calculator significantly improved the AUC for cancer detection (AUC = 0.7298; 95% CI, 0.7119 to 0.7478), but not for high-grade cancer (AUC = 0.7256; 95% CI, 0.7017 to 0.7495). The risk calculator also demonstrated an increased net benefit on decision curve analysis.

**Conclusion:**

The risk calculator developed has advantages over prior risk stratification of prostate cancer patients before the biopsy. It will reduce the number of men requiring a biopsy and their exposure to its side effects. The interactive tools developed are beneficial to translate the risk calculator into practice and allows for clarity in the clinical recommendations.

## Background

Prostate Cancer (PCa) is the most common non-cutaneous cancer in men in Ireland [1] and internationally [2]. This disease causes significant morbidity and mortality; every year over 500 men in Ireland die from this disease [3]. In fact, PCa is second only to lung cancer as the single most significant cause of cancer-specific mortality.

The current standard for the diagnosis of PCa is a prostate biopsy, the decision for which has classically been informed by an individual patient’s Prostate Specific Antigen (PSA) level in conjunction with Digital Rectal Examination (DRE). Unfortunately, the decision to proceed to prostate biopsy, an essential step in the accurate diagnosis of PCa lacks an appropriate sensitivity and specificity based on these parameters. This has led to the diagnosing clinically insignificant cancer and treatment of disease exposing a large number of men to unnecessary biopsies (false positives), anxiety about their diagnosis and treatment impacts on their quality of life. There are also a large number of men who are diagnosed with low-grade PCa (Gleason 6), who need not have been diagnosed, as their disease is unlikely to lead to an impact on their life span.

Accurate risk stratification of patients before biopsy would help to reduce the number of men going for a biopsy and thus overdiagnosis of insignificant disease and lead to better clinical decision making. Risk calculators (clinical prediction models) have long been in use within medicine to influence clinical decision-making. They mainly are models taking patient’s risk factors, combining them into an equation to assign a level of risk. Risk calculators can be used to predict many outcomes, be that success of a surgical procedure [4] or prognosis following acute myocardial infarction [5]. The level of risk can then be quantified as a percentage. In this way, risk calculators offer a logical and systematic approach to the use of patient risk factors to derive a percentage risk estimate.

In 2010, more than 100 prostate cancer risk calculators were published in various distinct populations [6], while a recent meta-analysis by Louie et al. found that 127 risk calculators existed [7]. One such risk calculator is the Prostate Cancer Prevention Trial (PCPT) risk calculator, which was created in a population of 5519 men from the United States, all of which were enrolled in the placebo arm of this randomised control trial. It was updated in 2014 to the PCPT 2.0. Data from over 1000 additional patients from the placebo arm of the original trial were added to form the PCPT 2.0 [8]. However, PCPT has been degraded and replaced by the Prostate Biopsy Collaborative Group (PBCG) in 2018 [9] since its lack of calibration in the modern practice. Another leading risk calculator was formed from the patients enrolled as part of the European Randomised Study of Screening for Prostate Cancer (ERSPC), which developed in European population to predict PCa using PSA, DRE, prostate volume and the previous negative biopsy status [10].

The ERSPC and the PCPT have been tested in an Irish population and proved to be beneficial [11] but further improvements in predictions need to be implemented. One approach would be to build the calculator in the relevant population with characteristics similar to that patient population [12]. This is especially important in the context of the Irish population, as Ireland currently operates a standardised referral programme through eight rapid access clinical around the country. This study aims to build an Irish risk calculator and compare it with PSA, the PCPT and ERSPC risk calculator and assessed using a number of performance indicators with the purpose to reduce the number of men going for a biopsy without missing significant cancer.

## Methods

### Study population

A national collection of patient information was undertaken to accumulate a database sufficient for the creation of an Irish prostate cancer risk calculator. The cohort consisted of 4801 biopsies performed between April 2010 and June 2015. Geographically distinct regions, even within countries, could have different patient populations. In order to capture these differences and attempt to risk stratify patients for prostate cancer in Ireland, it is imperative that men from each location within Ireland are used to create a clinical prediction model. Therefore, all patients were recruited from the eight Rapid Access Prostate Cancer Clinics, which have been put in place by the National Cancer Control Programme to expedite access to specialist Urologist care for men with suspected PCa. A consultant Urologist at each Rapid Access Prostate Cancer clinic then sees patients before the decision for a prostate biopsy is made. The shared decision to proceed to biopsy of the prostate is based on the patient history, DRE and a serum PSA level following informative discussion with the patient. This is important because it means that there was no strictly defined PSA threshold cut off value at which all men above this value were biopsied. Each patient underwent a systematic 12-core TRUS prostate biopsy, with additional cores being taken of suspicious echogenic lesions. The patient population was analysed histologically by the local consultant pathologist for a positive PCa diagnosis following biopsy and were subdivided according to Gleason grade, as defined by the International Society of Urological Pathology (ISUP) Consensus Conference 2005 [13]. Multiple Urologists and Pathologists may results in variation in decision-making and interobserver histological assessment highlighting the need for a central decision-making tool.

Patients attending these clinics had their data collected prospectively between 2010 and 2015. This was done to allow for reporting of critical parameters to the National Cancer Control Programme (NCCP) on a regular basis. A retrospective review was undertaken to expand upon each centre’s data to include relevant risk factors and to fill in missing data. This was performed using the individual’s hospitals computer systems for laboratory data, pathological results, radiology reports and basic clinical information.

### Statistical analysis

In order to perform descriptive statistics in this study cohort, patients were divided into those diagnosed with PCa on biopsy and those without a cancer diagnosis. The unpaired Student’s t-test and Wilcoxon Rank Sum test were used to examine the statistical significance of differences in means and medians between these two patient groups for continuous variables, while Pearson’s Chi-squared test was performed for categorical variables. Statistical analysis was performed in the R software version 3.4.3 [14].

The creation of an Irish risk calculator for the prediction of PCa and high-grade PCa utilized a generalised linear model by considering a complementary log-log link function for a binary logistic regression model. A stepwise selection method with a level of 5% for entry and a level of 10% for factor removal was applied to select the best clinical variables. The binary logistic regression was selected following a comparison with classification trees in terms of accuracy and is consistent with previous findings with the group [15]. In the binary logistic regression, the probabilities for each patient will be assigned through a function of risk factors. This function can then be converted to probabilities for each patient, and each can be assigned a percentage risk.

The Irish Prostate Cancer Risk Calculator (IPRC) for the diagnosis of PCa (and high-grade PCa) is built for the Irish population on the total cohort including linear and non-linear effects of components such as age, digital rectal examination, family history of PCa, prior negative biopsy and PSA level. Final models are illustrated in model summary tables as well as the corresponding nomograms which is a visual tool to calculate patient-relative risks along with simply displaying the variable importance. This calculator represents a ‘clinical’ risk calculator with all risk factors readily available. A second version of the IPRC was created in a subgroup of 2970 patients for whom prostate volume measurements were available and included prostate volume as an added risk factor. Each model underwent 10-fold cross-validation; this involves randomly dividing the data into ten evenly sized subgroups. A model is then constructed using the data from the first 9-folds and applied to the tenth group. The model building, and validation process are repeated ten times with each fold of patients used once as the validation set. This results in no patient being used to both develop and test the model. Internal validation of the PCa and high-grade PCa risk calculators took place following cross-validation and their performances were assessed using ROC analysis, calibration plots and decision curve analysis. The IPRC is compared to the PSA and PCPT 2.0 risk calculator.

Various graphical and numerical performance outcome summaries were used to demonstrate the discrimination ability of the model. The Receiver operating characteristic (ROC) curves and the decision curve analysis were used as standard graphical tools. Comparison of the ROC curves took place via a method described by DeLong et al. [16]. ROC analysis produces an area under the curve (AUC) for each model by plotting the sensitivity and specificity of the model at each of its risk thresholds. The AUC value along with sensitivity, specificity, Positive predictive value (PPV), Negative predictive value (NPV) and Youden index are utilised as numerical summaries which are shown to be beneficial when used in combination to each other. Calibration plots with Loess smoothing were generated to assess the agreement between the observed incidence of cancer and predicted risk [17]. Significance values for goodness of fit were computed using the Chi-Square Hosmer-Lemeshow test; for this test, a *p* < 0.05 indicates a poor agreement between the predicted risk and observed incidence.

Decision curves, which plot the net benefit of a model compared to the net benefit of a strategy of performing a biopsy on all patients or none, were formed as per Vickers et al. [18]. The area of the graph for which a risk calculator has a higher net benefit than both the ‘biopsy none’ and ‘biopsy all’ lines is where it has greatest clinical applicability. When comparing risk calculators, the model that occupies the greatest of this clinically applicable area and has the highest net benefit should be selected for clinical use.

The proposed model calculates the risk of having prostate cancer as a probability; however, in practice, an optimal probability (threshold) needed to be chosen to make the best clinical decision. The selection of this threshold could be challenging as it depends on a trade-off between a more sensitive test or a more specific test. A combination of various graphical summaries (i.e. sensitivity, specificity, PPV, NPV, Youden index) used to depict different aspects of discriminative ability of models on the threshold axis. Finally, an interactive web application is built to be presented to clinicians and decision makers which combine the graphical and numerical summarises to convey the result of the risk calculator in the most translated way.

## Results

The study cohort consisted of 4801 patient biopsies, and the characteristics of the full cohort are outlined in Table 1, and the characteristics of patients whom prostate volume has measured are given in Table 2. Of the total cohort, 2548 (53%) were diagnosed with PCa, while 1579 (33%) were diagnosed with high-grade PCa and Low-grade PCa (i.e. Gleason 6), represented 38% of all positive prostate biopsies. The most common score was Gleason 7, accounting for 42% of positive biopsies, while Gleason 8 and 9 accounted for 20% of positive biopsies while the rates of Gleason 10 diagnosis represented less than 1% of all detected cancers.

**Table 1:** Clinical Characteristics of all patients included in the Irish prostate cancer risk calculator study cohort

	All patients (*n* = 4801)			PCa patients (*n* = 2548)		
	PCa	No PCa	*P*-value	High grade PCa	Low grade PCa	*P*-value
**Patients**	2548 (53%)	2253 (47%)		1579 (62%)	969 (38%)	
**Median Age (mean)**	64.37 (63.70)	63.00 (62.28)	< 0.001 (< 0.001)	65.00 (64.40)	63.01 (62.57)	< 0.001 (< 0.001)
**Family history of PCa**						
Yes	182 (7%)	128 (6%)	< 0.001	114 (7%)	68 (7%)	0.173
Not recorded	1919 (75%)	1624 (72%)		1171 (74%)	748 (77%)	
No	447 (18%)	501 (22%)		294 (19%)	153 (16%)	
**Median PSA (mean)**	7.18 (18.87)	6.30 (7.31)	< 0.001 (< 0.001)	8.02 (26.04)	6.20 (7.17)	< 0.001 (< 0.001)
**DRE**						
Normal	1097 (43%)	1283 (57%)	< 0.001	555 (35%)	542 (56%)	< 0.001
Not recorded	359 (14%)	506 (22%)		210 (13%)	149 (15%)	
Abnormal	1092 (43%)	464 (21%)		814 (52%)	278 (29%)	
**Previous negative biopsy**						
Yes	354 (14%)	68 (20%)	< 0.001	152 (10%)	202 (21%)	< 0.001
No	2194 (86%)	1810 (80%)		1427 (90%)	767 (79%)	
**Gleason Score**						
Gleason 6	969 (38%)				969 (100%)	
Gleason 7	1058 (42%)			1058 (67%)		
Gleason 8	301 (12%)			301 (19%)		
Gleason 9	201 (8%)			201 (13%)		
Gleason 10	19 (< 1%)			19 (1%)

**Table 2:** Clinical Characteristics of patients whom their prostate volume is recorded

	All patients with prostate volume (*n =* 2970)			PCa patients with prostate volume (*n =* 1689)		
	PCa	No PCa	*P-*value	High grade PCa	Low grade PCa	*P*-value
**Patients**	1689 (57%)	1281 (43%)		1022 (61%)	667 (39%)	
**Median Age (mean)**	64.89 (64.05)	63.00 (62.00)	< 0.001 (< 0.001)	65.00 (64.68)	64.00 (63.09)	< 0.001 (< 0.001)
**Family history of PCa**						
Yes	129 (8%)	91 (7%)	0.741	83 (8%)	46 (7%)	0.192
Not recorded	1186 (70%)	894 (70%)		701 (69%)	485 (73%)	
No	374 (22%)	296 (23%)		238 (23%)	136 (20%)	
**Median PSA (mean)**	7.19 (19.45)	6.42 (7.45)	< 0.001 (< 0.001)	7.82 (27.43)	6.30 (7.23)	< 0.001 (< 0.001)
**DRE**						
Normal	735 (44%)	706 (55%)	< 0.001	361 (35%)	374 (56%)	< 0.001
Not recorded	241 (14%)	286 (22%)		143 (14%)	98 (15%)	
Abnormal	713 (42%)	289 (23%)		195 (51%)	195 (29%)	
**Previous negative biopsy**						
Yes	277 (16%)	303 (24%)	< 0.001	113 (11%)	164 (25%)	< 0.001
No	1412 (84%)	978 (76%)		909 (89%)	503 (75%)	
**Median Prostate volume (mean)**	35 (39.9)	45 (51.9)	< 0.001 (< 0.001)	34.1 (38.5)	37.3 (42.0)	< 0.001 (0.001)
**Biopsy Gleason Score**						
Gleason 6	667 (40%)				667 (100%)	
Gleason 7	659 (39%)			659 (64%)		
Gleason 8	222 (13%)			222 (22%)		
Gleason 9	124 (7%)			124 (12%)		
Gleason 10	17 (1%)			17 (2%)		

**Fig. 1:**
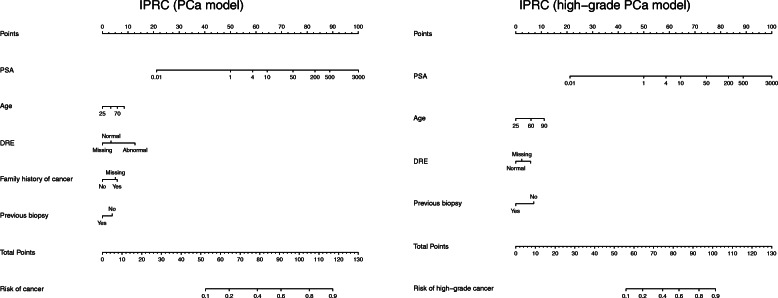
IPRC nomograms. The nomograms for PCa model is on the left, and high-grade PCa model on the right. The horizontal line on the top labelled `points’ allows the effect size of each variable to be assessed. To use the nomogram draw a straight line from the values/levels of each variable to measure its corresponding point. The total points on the bottom are then mapped to obtain the risk of cancer or high-grade cancer

The individual effects of all the risk factors were statistically significant in detecting cancer. This means that (on average) patients have more chance of prostate cancer if they are older, have higher PSA measured, have Abnormal DRE, have a family history of cancer or have not had a previous negative biopsy. Age, PSA, DRE and previous negative biopsy were also significant for predicting high-grade cancer; however, there was no significant individual effect of family history on detecting high-grade cancer. The IPRC models (PCa/high-grade PCa) are displayed in Table 3 and as two nomograms in Fig. 1.

**Table 3:** The IPRC models for predicting PCa on the left and high-grade PCa on the right. The coefficients, standard deviation and *p*-value represented for each variable in the logistic regression models

	IPRC – PCa model			IPRC – high-grade PCa model		
	Coefficients	Std. Error	*p-*value	Coefficients	Std. Error	*p-*value
**Intercept**	−2.100	0.202	< 0.001	−2.510	0.274	< 0.001
**Age**	0.009	0.003	0.005	0.016	0.004	< 0.001
**Family history (Positive)**	0.396	0.093	< 0.001	–	–	–
**Family history (Missing)**	0.344	0.056	< 0.001	–	–	–
**logPSA**	0.425	0.034	< 0.001	0.604	0.050	< 0.001
**DRE (Abnormal)**	0.639	0.046	< 0.001	0.560	0.060	< 0.001
**DRE (Missing)**	−0.230	0.063	< 0.001	0.228	0.0863	0.008
**Previous negative biopsy (Yes)**	−0.259	0.060	< 0.001	−0.679	0.091	< 0.001

**Fig. 2:**
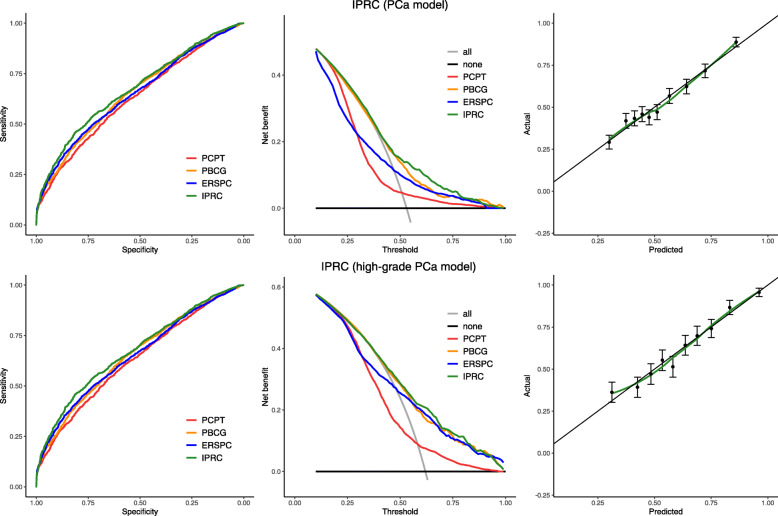
IPRC calibration and model comparison. The receiver operating characteristic (ROC) curves on the left and decision curves in the middle represent the discriminative ability of PCPT (red), PBCG (orange), ERSPC (blue) and IPRC (green) in diagnosis cancer (on top) and high-grade cancer (on the bottom). The calibration curves on the right indicate that predicted probabilities of both IPRC models are almost similar to the actual outcomes

The discriminative ability of the IPRC model compared to PSA (the current biomarker indicator), PCPT, PBCG and ERSPC in the prediction of both PCa and high-grade PCa is illustrated in Table 4. The Irish Model has shown an AUC of 0.67 for diagnosis of PCa and 0.72 for a high-grade PCa, which represented an improvement over PSA, PCPT, PBCG and ERSPC calculators for PCa diagnosis. The model for diagnosis of high-grade PCa was also outperformed other methods, although a non-significant AUC improvement over ERSPC. This is also visible from the ROC and decision curves in Fig. 2.

**Table 4:** The discriminative ability of PSA, PCPT, PBCG, ERSPC and IPRC using the areas under the curve (AUC) and 95% confidence interval of the calculated probabilities. The *p*-values indicate if the difference between each method and IPRC is significant

Models	Prostate cancer (*n* = 4801)			High grade cancer (*n =* 2548)		
	AUC	95% CI	*p-*value	AUC	95% CI	*p-*value
**PSA**	0.5948	0.5789–0.6107	*P* < 0.001	0.6623	0.6413–0.6832	*P* < 0.001
**PCPT**	0.6304	0.6148–0.6460	*P <* 0.001	0.6804	0.6597–0.7012	0. 005
**PBCG**	0.6528	0.6375–0.6681	*P* < 0.001	0.7185	0.6988–0.7381	0.839
**ERSPC**	0.6502	0.6349–0.6655	*P <* 0.001	0.7140	0.6942–0.7338	0.604
**IPRC**	0.6741	0.6591–0.6890	–	0.7214	0.7018–0.7409	–

Both IPRC model was well calibrated in this cohort (Fig. 2), with good agreement between predicted probabilities and the actual outcome. The Hosmer-Lemeshow test for goodness-of-fit shows that for the IPRC model in both PCa prediction (*p* = 0.09) and high-grade PCa prediction (*p* = 0.23) has satisfactory fit. Therefore, it can be trusted to classify patients into their risk groups.

Clinical utility, analysed via a decision curve, is also illustrated in Fig. 2. The decision curve analyses for diagnosis of cancer/High-grade cancer demonstrates higher net benefits compared to PCPT, PBCG and ERSPC calculators constantly. This means that if the IPRC is utilised there could be an increase in the diagnosis of PCa and/or a decrease in the number of unnecessary biopsies compared to the other methods, as was done in the patient population of the present study.

**Fig. 3:**
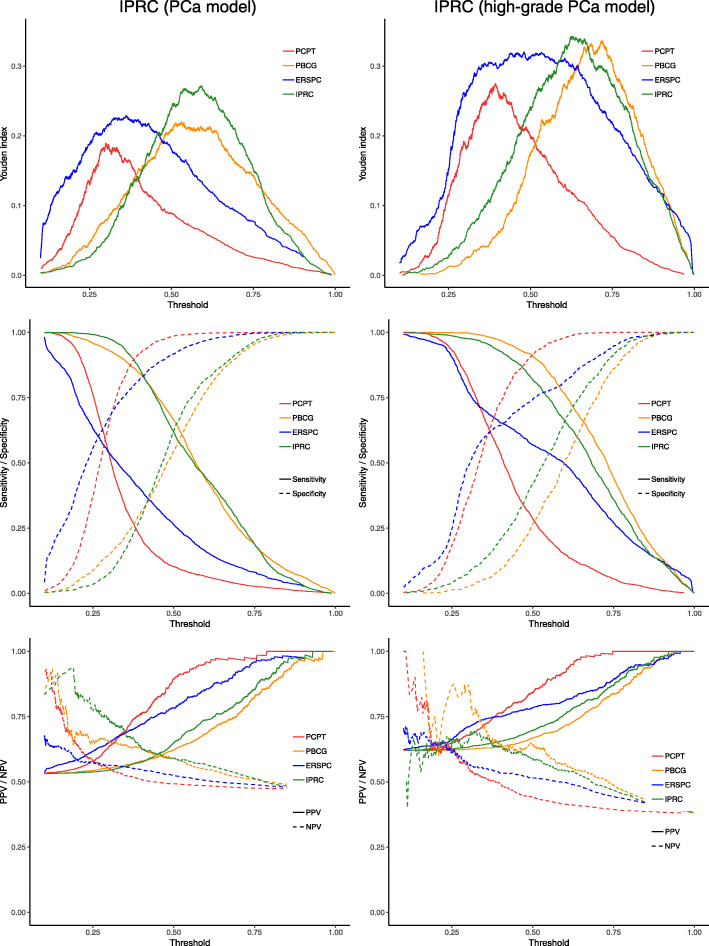
IPRC discrimination ability. Sensitivity, specificity, positive predictive value (PPV), negative predictive value (NPV), Youden index of the PCPT (red), PBCG (orange), ERSPC (blue) and IPRC (green) on the variously selected thresholds. The PCa model is displayed on the left and high-grade PCa model on the right

Figure 3 represents the discrimination ability of PCPT, PBCG, ERSPC and IPRC for diagnosis cancer/high-grade cancer across the different threshold. It also locates the best threshold ranges for the three risk calculators where PCPT and ERSPC suggest smaller cut-off points for both cancer / high-grade cancer diagnosis compared to PBCG and IPRC. The highest peak of the Youden index is about 0.27 for cancer diagnosis (threshold from 0.53 to 0.60 in IPRC) and about 0.34 for high-grade cancer diagnosis (threshold from 0.62 to 0.67 in IPRC), which shows the possibility of accessing more sensitive as well as more specific tests using IPRC. This ‘statistically suggested range’ could then be discussed with clinicians to select the best clinically accepted threshold to be used in practice. This range would provide flexibility in the clinical decision making to either increase the True positive rate (improve cancer detection) or decrease the False positive rate (reduce unnecessary biopsy). For this reason, a decision-making application [19] for IPRC (PCa diagnosis) which combines the graphical and numerical summarises is created to be presented to clinicians and decision makers to convey the result of this risk calculator in the most translated way.

Additionally, it is necessary to consider the corresponding sensitivity, specificity, PPV and NPV values to select the best clinically accepted threshold. A smaller threshold leads to a more sensitive test with the higher proportion of true negative results; however, a larger threshold leads to a more specific test with the higher proportion of correct positive results. Also, the wider horizontal line on the top of the Youden curve provides clinicians with more flexibility in the selection of a more sensitive or specific test. PCPT in both cases gives the narrowest ranges compared to ERSPC and IPRC. Although, the widest range relates to ERSPC in the diagnosis of high-grade cancer (from 0.35 to 0.64); however, IPRC is giving slightly better results in a smaller but still broad range (from 0.58 to 0.71).

**Fig. 4:**
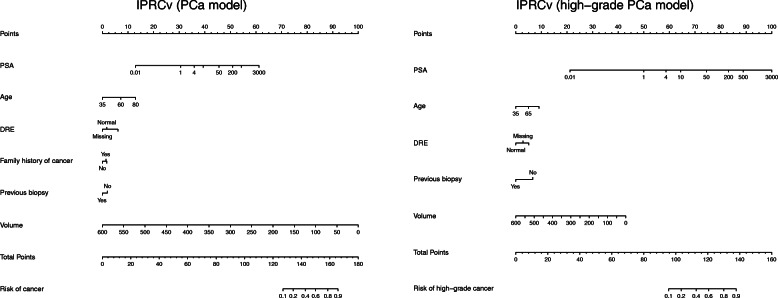
IPRCv nomograms. The nomograms (based on IPRCv which including prostate volume) for PCa model is on the left and high-grade PCa model on the right. The horizontal line on the top labelled `points’ allows the effect size of each variable to be assessed. To use the nomogram draw a straight line from the values/levels of each variable to measure its corresponding point. The total points on the bottom are then mapped to obtain the risk of cancer or high-grade cancer

The second version of Irish prostate cancer risk calculator including prostate volume (IPRCv) has built to incorporate prostate volume into the IPRC based on a subgroup of patients in whom TRUS volume was available (Table 1). New models (PCa/high-grade PCa including prostate volume) are displayed in Table 5 and as nomograms in Fig. 4.

**Table 5:** The IPRCv models for predicting PCa on the left and high-grade PCa on the right. The coefficients, standard deviation and *p*-value represented for each variable in the logistic regression models

	IPRCv – PCa model			IPRCv – high-grade PCa model		
	Coefficients	Std. Error	*p-*value	Coefficients	Std. Error	*p-*value
**Intercept**	−2.527	0.260	< 0.001	−2.369	0.342	< 0.001
**Age**	0.033	0.004	< 0.001	0.017	0.005	0.002
**Family history (Positive)**	0.158	0.111	0.152	–	–	–
**Family history (Missing)**	0.200	0.067	0.003	–	–	–
**logPSA**	0.435	0.045	< 0.001	0.650	0.065	< 0.001
**DRE (Abnormal)**	0.498	0.059	< 0.001	0.527	0.075	< 0.001
**DRE (Missing)**	−0.193	0.078	0.013	0.289	0.105	0.006
**Previous negative biopsy (Yes)**	−0.226	0.070	0.001	−0.693	0.106	< 0.001
**Prostate volume**	−0.019	0.001	< 0.001	−0.007	0.002	< 0.001

The discriminative ability of this model for diagnosis of both PCa and high-grade PCa over the model without the prostate volume on the same cohort is represented in Table 6. Including prostate volume significantly improved the AUC value of IPRC from 0.66 to 0.73 for the diagnosis of PCa, but it does not significantly improve the IPRC model for high-grade cancer. This demonstrated a significantly improved predictive ability over the European risk calculator including the prostate volume information (ERSPC-vol) for detecting cancer.

**Table 6:** The discriminative ability of PCPT, ERSPC and IPRCv using the areas under the curve (AUC) and 95% confidence interval of the calculated probabilities for those whom the prostate volume is available. The *p*-values indicate if the difference between each risk calculator and IPRCv is significant

Models	Prostate cancer (*n =* 2970)			High grade cancer (*n* = 1689)		
	AUC	95% CI	*p-*value	AUC	95% CI	*p-*value
**IPRC**	0.6597	0.6404–0.6790	< 0.001	0.7226	0.6986–0.7466	0.434
**ERSPC-vol**	0.6794	0.6604–0.6985	< 0.001	0.7176	0.6934–0.7419	0.174
**IPRCv**	0.7298	0.7119–0.7478	–	0.7256	0.7017–0.7495	–

IPRCv model could also be trusted to classify patients into their risk groups, since both models are well calibrated in this cohort and had satisfactory fit using goodness-of-fit test (*p* = 0.54 for PCa prediction and *p* = 0.82 for high-grade PCa prediction).

An interactive prediction application [20] based on the IPRC has been developed which estimates the risk of prostate cancer and high-grade cancer using the clinical variables. It includes an option to add the prostate volume which theoretically improves the accuracy of estimations. This user-friendly tool is not only accurately enhancing the risk stratification but also easily accessible to be used into daily urologic practice.

## Discussion

The accurate risk stratification of patients under investigation for PCa is of paramount importance. Indeed, PCa is not alone in this regard, as Nguyen and Kattan have remarked, “the ability to predict clinical outcomes accurately is critical to the proper management of any human disease” [21]. The IPRC demonstrated, in this cohort of Irish men, a superior performance in the prediction of prostate cancer and high-grade cancer. Importantly, the IPRC does not require any additional tests beyond those in current routine clinical practice. A PSA test will have already been performed before a patient is referred to a tertiary centre. The digital rectal examination is performed at these clinics, while all other risk factors can be obtained by questioning the patient. The Irish PCa risk calculator has the potential to improve the decision for prostate biopsy in the Irish clinical setting and could easily be introduced into routine practice. It would allow clinicians to apply a standardised and logical approach to the diagnosis of PCa and importantly, will provide a percentage risk that can be used simply to counsel each patient, facilitating a shared decision on prostate biopsy. This has been undertaken previously using the ERSPC risk calculator [22] and has been shown to be acceptable to patients. PCPT and ERSPC risk calculators are built on the outdated 6-core biopsies while PBCG and IPRC are both developed using 12-core biopsies which are more compatible with the modern practice. This might be the reason that IPRC demonstrated similar discrimination in Fig. 3 to the PBCG compared to PCPT and ERSPC.

In practice, the selection of an optimal threshold for the risk calculator would be as important as the risk calculator itself as a poor threshold selection could significantly affect the predictive ability of the risk calculator. The decision-making application created for threshold selection is an informative interactive tool for clinicians which aids the best decision making. A conservative decision strategy using two identified thresholds (0.25 and 0.5) used in the prediction application [20] to classify patients into ‘Low’, ‘Intermediate’ and ‘High’ risk groups. The threshold of 0.25 will ensure avoiding unnecessary biopsies for patients with very low risk of PCa while the threshold of 0.5 will suggest biopsy for high risk patients. The biopsy decision of patients with intermediate risk can be made individually by considering other factors such as patients’ life expectancy or patients’ preference.

Since confirmed previously [11, 23] the use of prostate volume estimate (either TRUS volume estimates or DRE volume estimates) improved the discriminative ability of the risk stratification. Regardless of which IPRC is selected, the ‘clinical’ model or that containing a prostate volume estimate, this risk calculator may prove useful to identify suitable patients for MRI pre-biopsy. PCa remains one of the few malignancies diagnosed using a non-targeted approach to biopsy, although this paradigm is rapidly changing [24]. Despite the increasingly common practice of MRI-guided biopsy, risk calculators can be utilised in clinical practice in order to stratify patients for MRI and to direct this resource towards those patients most suitable.

The strength of this study lies in the large numbers of Irish patients that have been collected and the multi-institutional design. However, this risk calculator can be improved upon using individual risk factors, which is the main avenue towards the improvement of this Irish Model. Novel biomarkers can improve upon PSA [25, 26], DRE volume estimation can be utilised [10], and family history can be recorded in a systematic fashion. In particular, family history had a significant effect on the diagnosis of cancer but not high-grade cancer. However, it has been poorly recorded in this cohort. Efforts should be made to record each patient’s family history carefully, and precisely the age at PCa diagnosis and the aggressiveness of the PCa diagnosed in order to make the best use of the basic clinical information available to us. Furthermore, Grill et al. have demonstrated in a recent paper that family history adjusted for age at diagnosis is a significant independent risk factor for PCa [27].

There are several limitations to this body of work. The inherent limitation of this study, and others like it, is the possibility of a false negative biopsy result. This risk has been reported in the literature to be as high as 24% [28]. If we consider these false negative results, then flaws will exist in the creation of any risk assessment tool that stratifies patients according to the result of their prostate biopsy. However, the approach discussed here to select a clinically accepted threshold that could help to control this error. Also, the patients belong to the ‘statistically suggested range’ could also be classified to require more consideration before undertaking an (invasive) prostate biopsy. Although our results demonstrate a significantly higher predictive accuracy compared to other methods in the Irish population, the new models have not been independently validated. Our group plans to externally validate this calculator using newly acquired patient biopsy information – which will allow for continuous updating as per Strobl et al. [29]. The lack of PSA standardisation throughout the country also limits the interpretation of this study’s results. PSA is the most important biomarker for the diagnosis of PCa within Ireland currently, and its measurement in Ireland is not yet standardised. The latest figures indicate that PSA testing within Ireland takes place in 37 distinct laboratories, with a 100-fold variation in workload among them. PSA levels have also been shown to have considerable variation from lab-to-lab in Ireland, and patient risk stratification is restricted by these variations [30].

A conceivable criticism of the present study is that it lacks broad applicability to a number of countries. However, this research was driven by the hypothesis that a calculator built in a foreign population would not perform as well as an Irish-specific tool. We believe the national multi-institutional approach to the creation of an Irish risk calculator is a core strength of this study.

## Conclusion

This study demonstrates that patient risk stratification for PCa can be improved within the Irish population through the use of multivariable risk assessment. A logical and standardised approach to the use of clinical risk factors can allow for more accurate risk stratification of men under investigation for PCa. We have developed a PCa risk calculator for the Irish population which can better inform the decision to perform a prostate biopsy. It could reduce the number needing a biopsy without impacting on the detection of cancer or significant disease, which represents an important impact on men by lowering their exposure to the side effects of biopsy as well as having to deal with the associated morbidity. However, the novel static/interactive graphical tools presented play a crucial role in selecting a reasonable threshold to use in practice.

## Data Availability

All data referenced and analysed come from the eight Rapid Access Prostate Cancer Clinics in Ireland, which were used under license for the current study, and so are not publicly available.

## Abbreviations

PCa: Prostate cancer
IPRC: Irish Prostate Cancer Risk Calculator
PSA: Prostate-specific antigen
PCPT: Prostate Cancer Prevention Trial
PBCG: Prostate Biopsy Collaborative Group
ERSPC: European Randomized Study of Screening for Prostate Cancer
IPRCv: Irish prostate cancer risk calculator including prostate volume
DRE: Digital Rectal Examination
ISUP: International Society of Urological Pathology
NCCP: National Cancer Control Programme
ROC: Receiver operating characteristic
AUC: Area under the curve
PPV: Positive predictive value
NPV: Negative predictive value
ERSPC-vol: European risk calculator including the prostate volume information
